# OFD1 and Flotillins Are Integral Components of a Ciliary Signaling Protein Complex Organized by Polycystins in Renal Epithelia and Odontoblasts

**DOI:** 10.1371/journal.pone.0106330

**Published:** 2014-09-02

**Authors:** Stephanie Jerman, Heather H. Ward, Rebecca Lee, Carla A. M. Lopes, Andrew M. Fry, Mary MacDougall, Angela Wandinger-Ness

**Affiliations:** 1 Department of Pathology MSC08-4640 and Cancer Research and Treatment Center, University of New Mexico Health Sciences Center, Albuquerque, New Mexico, United States of America; 2 Department of Internal Medicine, Division of Nephrology MSC10-5550, University of New Mexico Health Sciences Center, Albuquerque, New Mexico, United States of America; 3 Department of Biochemistry, University of Leicester, Leicester, United Kingdom; 4 Institute of Oral Health Research & Department of Oral and Maxillofacial Surgery, School of Dentistry, University of Alabama, Birmingham, Alabama, United States of America; Boston Children's Hospital Harvard Medical School Pediatrics, United States of America

## Abstract

Mutation of the X-linked oral-facial-digital syndrome type 1 (OFD1) gene is embryonic lethal in males and results in craniofacial malformations and adult onset polycystic kidney disease in females. While the OFD1 protein localizes to centriolar satellites, centrosomes and basal bodies, its cellular function and how it relates to cystic kidney disease is largely unknown. Here, we demonstrate that OFD1 is assembled into a protein complex that is localized to the primary cilium and contains the epidermal growth factor receptor (EGFR) and domain organizing flotillin proteins. This protein complex, which has similarity to a basolateral adhesion domain formed during cell polarization, also contains the polycystin proteins that when mutant cause autosomal dominant polycystic kidney disease (ADPKD). Importantly, in human ADPKD cells where mutant polycystin-1 fails to localize to cilia, there is a concomitant loss of localization of polycystin-2, OFD1, EGFR and flotillin-1 to cilia. Together, these data suggest that polycystins are necessary for assembly of a novel flotillin-containing ciliary signaling complex and provide a molecular rationale for the common renal pathologies caused by OFD1 and PKD mutations.

## Introduction

Oral-facial-digital syndrome type 1 (OFD1; OMIM #311200) is an X-linked inherited disease characterized by the malformation of the face, oral cavity, hands and feet caused by heterogeneous mutations in the OFD1 gene also known as CXORF5. Systemic manifestations of OFD1 mutations include polycystic kidneys that resemble those caused by mutations in the PKD1 or PKD2 genes associated with autosomal dominant polycystic kidney disease (ADPKD) [1], [2]. Due to the low rate of kidney transplantation, many patients with both craniofacial disorders and cystic kidney disease will eventually succumb to renal failure. Thus, there is an urgent need to clarify how the OFD1 gene product might cross-talk with the pathways regulated by the PKD1 and PKD2 genes to result in a common disease phenotype.

Many of the proteins associated with cystic kidney disorders, including polycystin-1 (PC1) and polycystin-2 (PC2) that underlie ADPKD, localize to and function in the primary cilium [3]–[7]. The polycystins have pivotal roles in calcium dependent signaling to multiple pathways and loss of signaling regulation when the proteins are mutant is thought to cause epithelial cell transdifferentiation and contribute to renal cyst development [2]. While the associations between defects in primary cilia, signaling and kidney disease have been recognized for over a decade, it is only recently that links between cilia, signaling, and tooth defects were revealed. Deletion of the Ofd1 gene (Ofd1Δ4-5/+-) in mice causes missing/supernumerary teeth, enamel hypoplasia, and polycystic kidney disease analogous to human oral-facial-digital syndrome type 1 [4]. The observed morphological defects in molars result from altered differentiation and polarization of odontoblasts when Ofd1 is mutant [3], [4], [8]. Localization of OFD1 to the primary cilium of tooth ectomesenchymal odontoblasts and renal epithelial cells is therefore speculated to be crucial for proper cellular differentiation of both cell types [3], [4].

In related observations in the Tg737 mouse, ectopic teeth (premolars normally evolutionarily silenced) arise from inactivation of IFT88/polaris in the embryonic jaw and the consequential increase in sonic hedgehog signaling [9]. The Tg737 mouse was first used to illuminate the central role of IFT88/Polaris in the development of cystic kidneys [10]. The emerging molecular hierarchy requires OFD1 to recruit IFT88 as a prerequisite for ciliogenesis and ciliary hedgehog signaling that also involves the polycystins [11]–[13]. Collectively, these findings hint that OFD1 and the polycystins are likely part of the same or overlapping protein assemblies that control similar ciliary signaling pathways in both odontoblasts and renal epithelia.

There are some striking similarities in the mechanism by which odontoblasts and renal epithelial cells respond to external stimuli with the primary cilium of odontoblasts believed to closely mimic the sensory function of cilia in renal epithelial cells [14]; thus, the primary cilium likely serves as the critical link between extracellular mechanical stimuli and initiation of responding intracellular signaling cascades in odontoblasts and renal epithelial cells. In addition to altered Hedgehog signaling, aberrant expression and signaling from the tyrosine kinase epidermal growth factor receptor (EGFR) is implicated in both craniofacial disorders and ADPKD [15]–[17], and in primary cilia of renal epithelia, EGFR interacts with and regulates PC2 ion channel activity [7]. Collectively, these data demonstrate the need to characterize the functional interactions and molecular assemblies of complexes comprised of signaling receptors, domain-organizing proteins, and ciliary proteins, which when mutant, cause similar disease phenotypes. We therefore tested the hypothesis that OFD1 co-assembles into protein complexes constituted of PC1 and PC2, EGFR, and the flotillin lipid scaffolding proteins in the primary cilium of renal epithelia and odontoblasts. Our findings provide a molecular explanation for some of the observed commonalities in the pathogenesis of multi-organ ciliopathies such as OFD1 syndrome and ADPKD.

## Results

### Expression of OFD1, polycystins, flotillins and ErbB receptor family members in renal epithelia and odontoblasts

We suspected the membrane raft organizing flotillin proteins [18], [19] to be key players in the organization of ciliary signaling complexes. The speculation is founded on our studies that first identified a cholesterol-rich, flotillin-organized signaling domain – constituted of polycystins, tyrosine kinases and phosphatases and cholesterol – at the basolateral membrane of renal epithelia [20], [21]. Of interest is the recent finding that EGFR and downstream signaling partners are scaffolded in cholesterol-rich signaling domains by the flotillins [22]. Here, we tested if OFD1 is co-assembled into protein complexes constituted of PC1 and PC2, EGFR and the flotillin lipid scaffolding proteins in the primary cilium of renal epithelia and odontoblasts.

**Figure 1:**
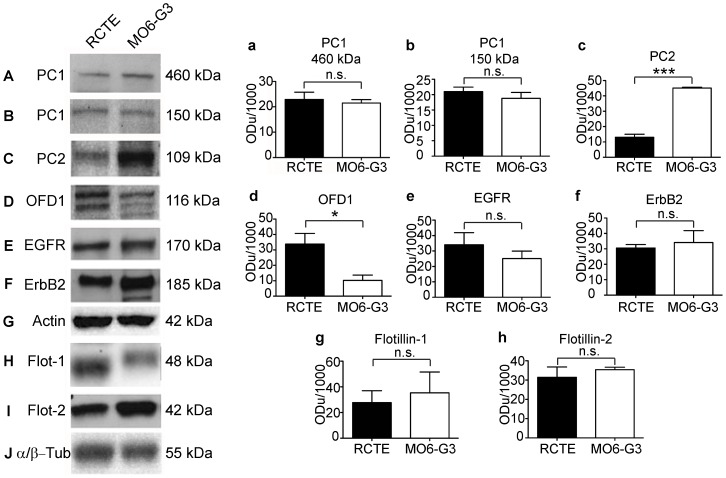
Key ciliary signaling proteins are expressed in RCTE and MO6-G3 cells. Renal cortical tubular epithelial (RCTE) cells and tooth derived odontoblasts (MO6-G3) cells were lysed and probed with antibodies directed against indicated proteins. PC1 using NM002 pAb (A, B), PC2 using Santa Cruz pAb (C), OFD1 using Santa Cruz pAb [3] (D), EGFR using Santa Cruz pAb (E), ErbB2 using US Biological pAb (F), flotillin-1 using BD Transduction mAb (H), and flotillin-2 using BD Transduction mAb (I) were found to be expressed in both cell types. Actin mAb from Millipore (G) was used as a loading control for PC1, PC2, EGFR, ErbB2, and OFD1; α/β-tubulin pAb from Cell Signaling (J) was used as a loading control for flotillin-1 and flotillin-2. Actin or α/β-tubulin was used to normalize results for quantification. Bar graph showing densitometric quantification of PC1 (a, b), PC2 (c), OFD1 (d), EGFR (e), ErbB2 (f), flotillin-1 (g), flotillin-2 (h). Bar graph represents the mean ± SD of four independent experiments. (*) p = 0.01 to 0.05, (***) p<0.001.

The expression of Erythroblastic Leukemia Viral Oncogene (ErbB) receptor proteins and the flotillins in renal epithelia and odontoblast cells is not well described. The expression of these and cystic kidney disease proteins were therefore subjected to comparative immunoblot analyses on polarized renal cortical tubular epithelial (RCTE) cells and odontoblasts (MO6-G3). PC1 (Figure 1A, B), PC2 (Figure 1C) and OFD1 (Figure 1D), along with the tyrosine kinase receptors EGFR (Figure 1E) and ErbB2 (Figure 1F) were robustly expressed in both human renal epithelial cells and mouse odontoblasts in culture. The signaling domain organizing proteins flotillin-1 (Figure 1H) and flotillin-2 (Figure 1I) were similarly expressed in both cell types, although flotillin-1 exhibited a clear mobility shift in odontoblasts as compared to renal epithelial cells. The native 460 kDa PC1 is known to be cleaved in cells yielding several different C-terminal fragments that are important for physiologic functions [6], [23], [24]. (N.B. Available antibodies to PC1 recognize different epitopes with some recognizing the full-length 460 kDa protein while other antibodies preferentially recognize the 230–250 kDa or 150 kDa PC1 bands).

Renal epithelia and odontoblasts expressed similar levels of both the 460 kDa and the 150 kDa forms of PC1 (Figure 1a, b), along with EGFR (Figure 1e), ErbB2 (Figure 1f), flotillin-1 (Figure 1g), and flotillin-2 (Figure 1h). Notably, protein expression levels of PC2 were 3.4-fold higher (Figure 1c), while OFD1 levels were 3.8 fold lower in odontoblasts as compared to renal epithelia (Figure 1d). These data show that key mechanosensory and signaling proteins such as the polycystin proteins, OFD1, EGFR, and the flotillins are common to both renal epithelia and odontoblasts, though there are some variations in the quantitative levels of the cation channel PC2 and OFD1.

### Colocalization of OFD1, polycystins, EGFR and flotillins to the primary cilium of odontoblasts and renal epithelial cells

The primary cilium has a distinct protein composition compared to the apical surface from which it protrudes, and is enriched in signaling proteins that also function at the basolateral membrane [25], [26]. The importance of the primary cilium as a signaling command center has been suggested [7], [27], yet little is known about the organization of signaling proteins within primary cilia.

**Figure 2:**
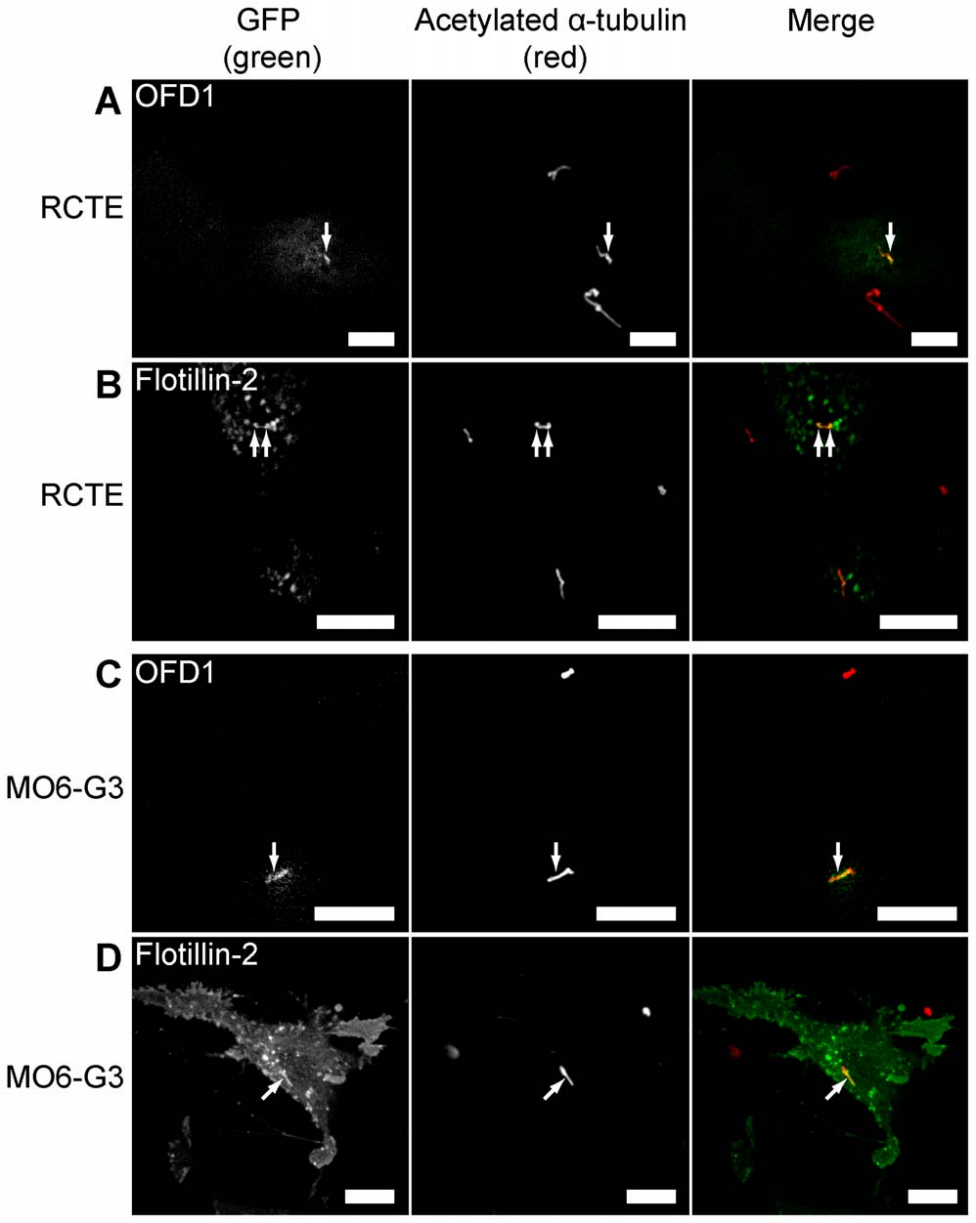
Transiently transfected GFP-OFD1 and flotillin-2-GFP localize to primary cilia of RCTE and MO6-G3 cells. Polarized RCTE and MO6-G3 cells were transiently transfected with either GFP-OFD1 or flotillin-2-GFP constructs. Upon 16 hours post-transfection, cells were fixed and acetylated α-tubulin (Sigma Aldrich mAb) was labeled to identify cilia. GFP-OFD1 localized to primary cilia in RCTE cells (A) and MO6-G3 cells (C). GFP-Flotillin-2 localized to primary cilia of RCTE (B) and MO6-G3 (D) cells. Images captured using a Zeiss LSM510 confocal microscope (63× objective). Images are representative of at least 3 independent experiments. Arrows denote cilia. Scale bar 10 µm.

Immunolocalization studies of endogenous and overexpressed proteins were performed to specifically assess ciliary protein localization patterns in odontoblasts and renal epithelial cells. Odontoblast cells and fully polarized RCTE cells are notoriously difficult to transfect via conventional techniques. To circumvent this problem, we found electroporation (iPorator) on filter surfaces routinely yielded plasmid transfection efficiencies of 40–50% (see Methods). Transiently expressed GFP-OFD1 (Figure 2A) and flotillin-2-GFP (Figure 2B) localized to primary cilia in RCTE cells with some flotillin-2 also on intracellular structures. Similarly, GFP-OFD1 (Figure 2C) and flotillin-2-GFP (Figure 2D) localized to primary cilia of odontoblast cells, though there were significant pools of flotillin-2-GFP elsewhere in the plasma membrane and intracellular structures. OFD1 is known to be associated with basal bodies and, in some renal cells, to localize along the ciliary axoneme [3]. Here we found that, particularly in odontoblasts, GFP-OFD1 staining extended beyond the region expected to contain just the basal bodies into the ciliary axoneme.

**Figure 3:**
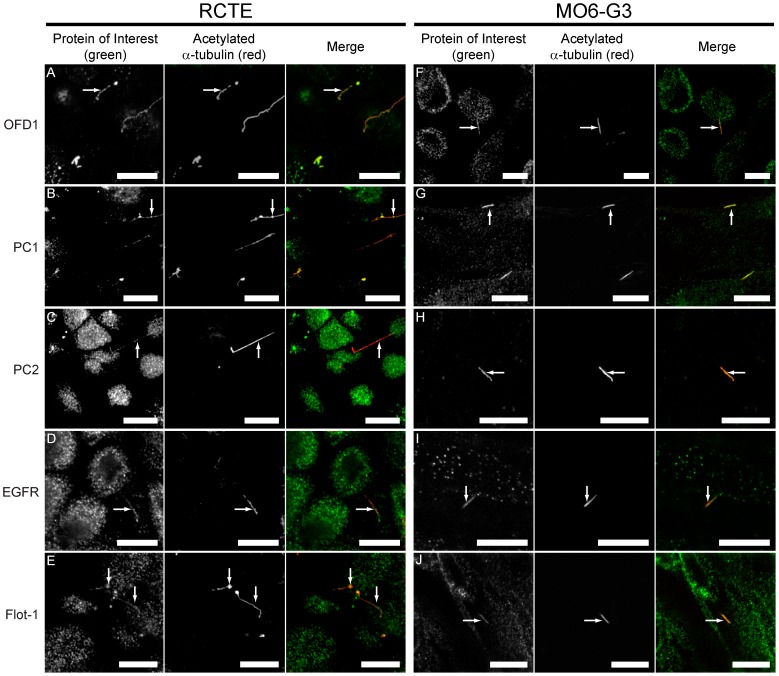
Endogenous OFD1, PC1, PC2, EGFR and flotillin-1 localize to primary cilia of RCTE and MO6-G3 cells. Polarized RCTE and MO6-G3 cells were fixed and stained using the indicated antibodies. OFD1 from Novus Biologicals (A, F), PC1 using pAb NM002 (B, G), PC2 using AbCam pAb (C, H), EGFR using GeneTex pAb (D, I) and flotillin-1 using AbCam pAb (E, J) were found localized to 100% of primary cilia analyzed for both cell types. Acetylated α-tubulin (Sigma-Aldrich mAb) labeling identifies cilia. Zeiss LSM510 confocal microscope images (63× objective). Arrows denote the protein of interest within a cilium. Representative results from at least 5 independent experiments. Secondary antibody only controls were negative (not shown). Scale bar 10 µm.

Endogenous protein localizations were evaluated by immunofluorescence staining using antibodies specifically directed against OFD1, PC1, PC2, EGFR, and flotillin-1. In RCTE cells OFD1, PC1, PC2, and EGFR localized along the length of the primary cilium (Figure 3A–D). The ciliary localization of flotillin-2-GFP prompted us to look for the flotillin-2 scaffolding partner, flotillin-1. As expected, endogenous flotillin-1 also localized to primary cilia in RCTE cells (Figure 3E). Similarly, we observed endogenous OFD1, PC1, PC2, EGFR, and flotillin-1 protein localization to primary cilia in odontoblast cells (Figure 3F–J). In RCTE cells, we observed co-localization of transiently expressed Flotillin-2-GFP and PC2 in primary cilia (Figure S1A) as well as Flotillin-2-GFP and EGFR (Figure S1B), while endogenous EGFR and PC1 co-localized to primary cilia of RCTE cells (Figure S1C). The ciliary localizations of OFD1, PC1, PC2 and EGFR are consistent with previous reports showing individual proteins in different cell types [3]–[7]. However, we show for the first time the localization of all 5 proteins, including the membrane organizing flotillins, to primary cilia in a human kidney cell line. The findings that ciliary protein distributions are similar in renal epithelia and odontoblasts suggest that these proteins may be similarly organized in both cell types.

To support the microscopy data, polarized RCTE cells were deciliated using dibucaine-HCl and immunoblot analyses performed on the ciliary enriched fraction. The ciliary marker alpha/beta-tubulin was expressed in abundance within the collected fraction, whereas the nuclear marker lamin B was absent. Given that the nuclear membrane is often tightly coupled to cilia via a centrosomal anchoring mechanism, the data support the ciliary enrichment in the absence of nuclear contamination of the cell fraction. PC1, PC2, and EGFR were all co-enriched within the ciliary fraction of RCTE cells providing further evidence for the presence of signaling proteins within primary cilia (Figure S2).

### Polycystins and OFD1 form protein complexes with EGFR and flotillins in odontoblasts and renal epithelial cells

**Figure 4:**
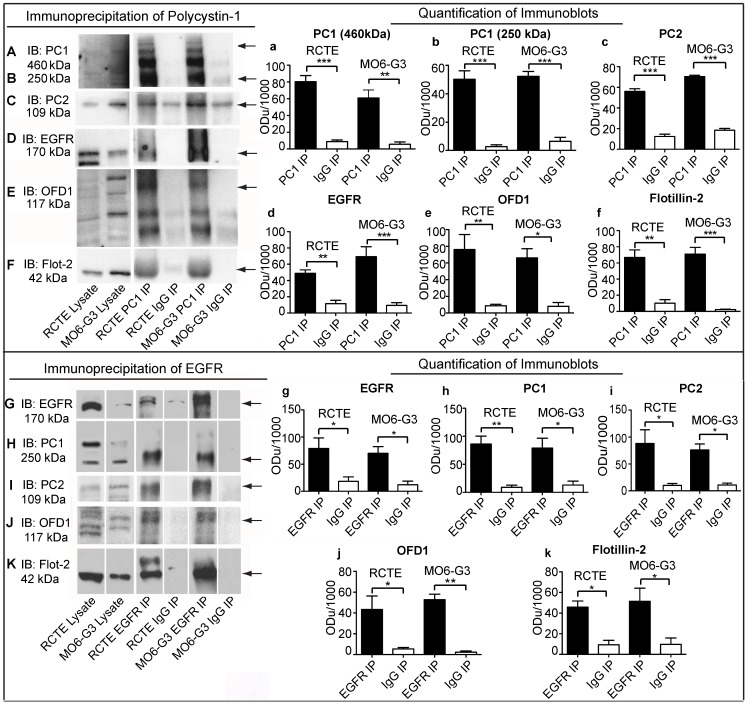
PC1, PC2, EGFR, OFD1, and flotillin-2 are part of a multimeric protein complex in RCTE and MO6-G3 cells. RCTE and MO6-G3 cells were grown 5 days post-confluency to allow for polarization and ciliogenesis. PC1 was immunoprecipitated using NM002 pAb and precipitated proteins were separated by SDS-PAGE and probed for PC1 using NM005 pAb (A, B), PC2 using AbCam pAb (C), EGFR using GeneTex pAb (D), OFD1 using Novus Biologicals pAb (E) and flotillin-2 using Cell Signaling Rabbit mAb (F). Bar graph showing a densitometric quantification of PC1 co-immunoprecipitation for PC1 (a, b), PC2 (c), EGFR (d), OFD1 (e), and flotillin-2 (f). Normal rabbit IgG was used as a negative control. In a reciprocal experiment EGFR was immunoprecipitated using Santa Cruz pAb from RCTE and MO6-G3 cell lysates. Immunoprecipitated proteins were separated by SDS-PAGE and probed for EGFR using GeneTex pAb (G), PC1 using NM002 pAb (H), PC2 using Santa Cruz pAb (I), OFD1 using Novus Biologicals pAb (J) and flotillin-2 using Cell Signaling Rabbit mAb (K). Bar graph showing a densitometric quantification of EGFR co-immunoprecipitation probed for EGFR (g), PC1 (h), PC2 (i), OFD1 (j), and flotillin-2 (k). Normal rabbit IgG was used as a negative control. Lysate lane inputs were 5% of immunoprecipitations. 25 µg of total protein was loaded into each well. Arrows indicate the quantified band of interest in each immunoblot panel. Bar represents the mean ± SD of at least three independent experiments. (*) p = 0.01 to 0.05, (**) p = 0.001 to 0.01, (***) p<0.001.

To determine whether the ciliary proteins of interest are part of a multimeric complex we performed immunoprecipitation experiments with specific antibodies against PC1 and EGFR and scored for co-precipitation of the proteins of interest. RCTE and odontoblast cells were grown to five days post-confluency to allow for polarization and ciliogenesis. PC1 was immunoprecipitated from renal epithelial and odontoblast cell lysate (Figure 4A) and shown to be in complex with PC2 as expected (Figure 4C). The native 460 kDa PC1 contains a region with potential metalloprotease recognition sequences yielding 230–250 kDa C-terminal fragments [6], [28], which were also co-precipitated (Figure 4B). PC1, along with PC2, becomes tyrosine phosphorylated potentially through the actions of EGFR [7], [21], [29]. To test if EGFR is part of the polycystin complex, PC1 immunoprecipitates were probed for EGFR revealing that EGFR was indeed in complex with PC1 in both cell types (Figure 4D). The non-transmembrane, ciliary protein OFD1 was also found to be part of the polycystin-EGFR multimeric complex in renal epithelia and odontoblast cells (Figure 4E), and flotillin-2 is in complex with PC1 in both cell types (Figure 4F). Quantification of replicate experiments demonstrated specific co-precipitation of PC2, EGFR, OFD1, and flotillin-2 with PC1 (Figure 4a–f). In a converse experiment, EGFR was immunoprecipitated from polarized renal epithelial and odontoblast cell lysates and probed for PC1, PC2, OFD1, and flotillin-2 (Figure 4G–K). As seen in the PC1 immunoprecipitates, all of the proteins were co-enriched with EGFR (Figure 4g–k), with a cleaved fragment of PC1 preferentially co-precipitated with EGFR. Multiple OFD1 bands are present in RCTE and MO6-G3 cell lysates and a subset of these OFD1 bands are co-precipitated with EGFR. Interestingly, the flotillin-2 bands were notably upshifted in both the PC1 and EGFR immunoprecipitates suggesting posttranslational modification within these complexes. These data indicate that key ciliary signaling proteins – PC1, PC2, EGFR and OFD1 – may well reside in a complex with membrane organizing flotillin proteins in renal and dental cell types.

### Polycystin-1 and EGFR form a complex within primary cilia

**Figure 5:**
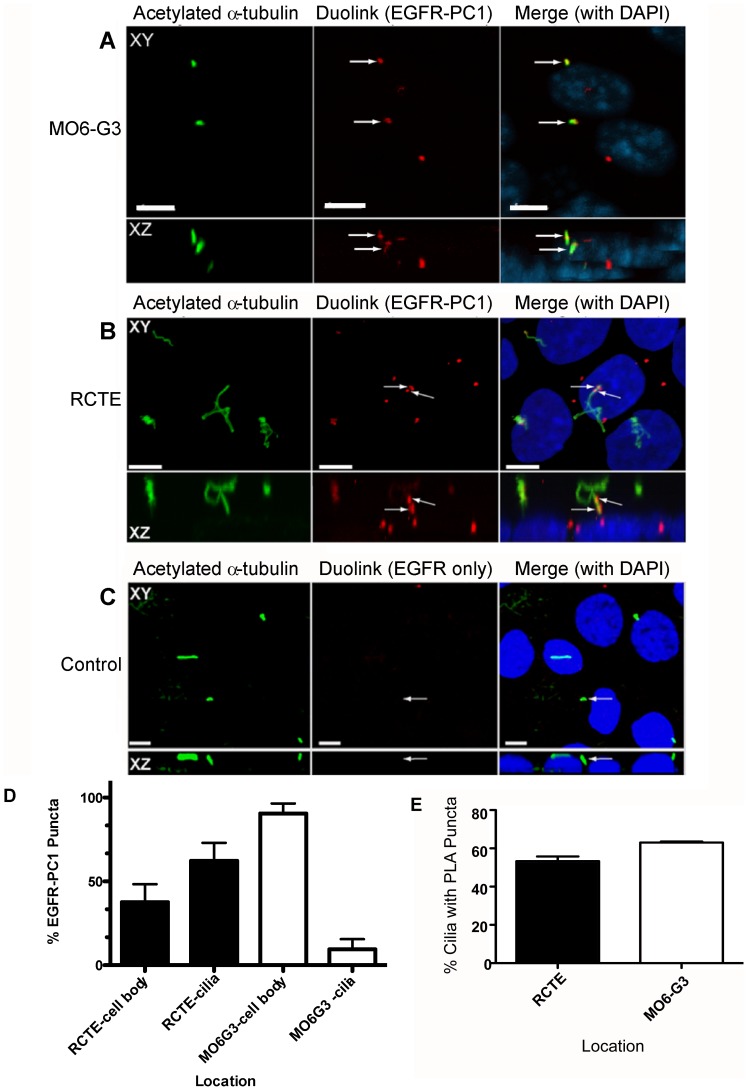
PC1 and EGFR interact in the primary cilium of MO6-G3 and RCTE cells. Polarized MO6-G3 (A) and RCTE (B) cells were grown on coverslips 5 days post-confluency. Cells were incubated with antibodies against PC1 (Santa Cruz mAb) and EGFR (GeneTex pAb), followed by Duolink PLA Probes to identify points of PC1-EGFR interaction. Cilia identified by acetylated α-tubulin (Sigma-Aldrich mAb). Identical experiments performed without PC1 antibody are negative for fluorescent signal (C). Top panel shows XY plane, bottom panel shows XZ plane. Images from confocal microscope (63× objective). Scale bar is 5 µm. Quantification of puncta in 3 representative images (11–29 cells/field) from 2 independent experiments (D). Quantification of cilia containing PLA puncta (E). Puncta in cilia were counted based on colocalization with acetylated α-tubulin. Statistical evaluation based on two-tailed t-test. p = 0.0194.

We utilized an antibody-based proximity ligation assay to specifically evaluate if the protein complexes we identified by co-immunoprecipitation were resident in cilia [30]. Using antibodies directed against the extracellular domains of PC1 and EGFR, these two proteins were found to interact specifically within the primary cilium of odontoblasts (Figure 5A) and fully polarized renal epithelial cells (Figure 5B and Movie S1). In both cell types the fluorescent signal was notably concentrated in puncta, suggestive of specialized domains wherein the proteins are enriched. In the relatively short cilia of odontoblasts, a less concentrated signal was also seen along the length of the shaft. Puncta associated with the cell body were largely intracellular with a small fraction at or near the basolateral membrane (Figure 5A–B and data not shown). Experiments performed without primary antibody against PC1 yielded no fluorescent signal verifying the specificity of the PC1-EGFR reaction (Figure 5C).

Quantification of these proximity-induced fluorescent puncta showed an enrichment in the cilia (62%) as compared to the cell body (38%) of fully polarized RCTE cells (Figure 5D). The pattern in odontoblasts was reversed with a greater fraction of puncta in the cell body (90%), yet retaining a quantifiable fraction in cilia (10%) (Figure 5D). Both cell types had similar numbers of ciliated cells (RCTE 82%; MO6-G3 93%), although the average cilia length was different (RCTE 6.10 µm; MO6-G3 0.49 µm). Both RCTE and MO6-G3 had a similar percentage of primary cilia containing puncta: RCTE 53.4%; MO6-G3 62.6% (Figure 5E). Taken in conjunction with previous data showing PC1 and PC2 interact in primary cilia [11] and PC2 forms a ciliary signaling complex with EGFR [7], these data indicate that PC1 and PC2 likely form a three way complex with EGFR in cilia.

### Mutant PC1 results in decreased ciliary localizations of PC2, OFD1, EGFR and flotillin-1

**Figure 6:**
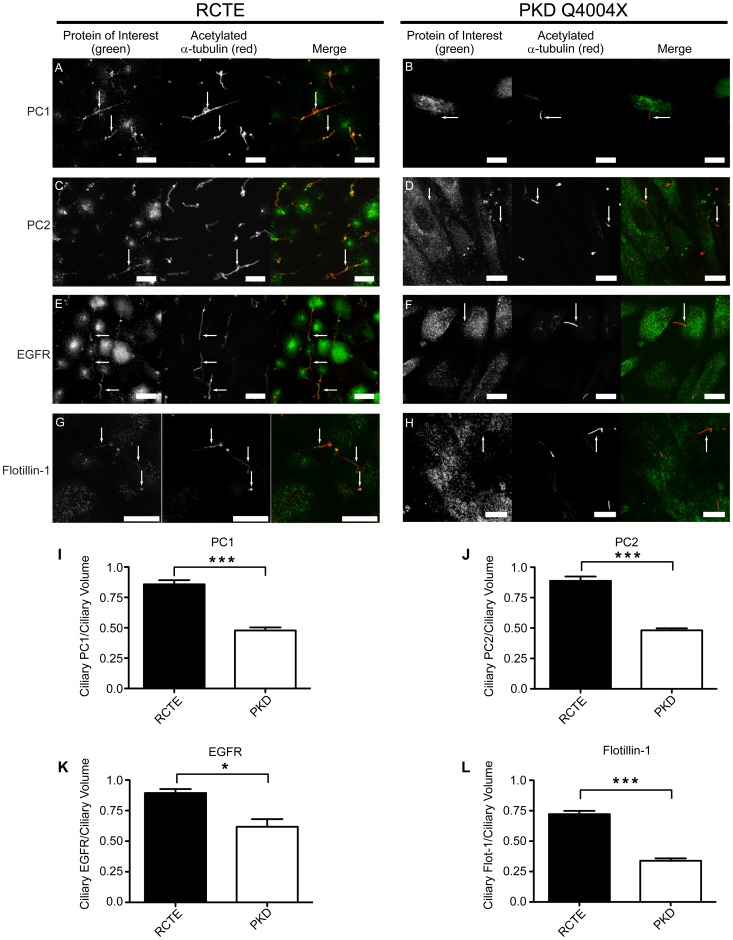
Key ciliary signaling proteins are significantly reduced in primary cilia of PKD cells. Human RCTE and PKD Q4004X cells (with expression of mutant PC1) were grown on coverslips 5 days post-confluency to permit ciliogenesis. Cells were fixed and stained using antibodies directed against indicated proteins: PC1 (NM002 pAb); PC2 (AbCam pAb); EGFR (GeneTex pAb); and flotillin-1 (AbCam pAb). PC1 (A), PC2 (C), EGFR (E) and flotillin-1(G) are present in primary cilia of RCTE cells. PC1 (B), PC2 (D), EGFR (F) and flotillin-1 (H) were lacking in primary cilia of PKD Q4004X cells. Acetylated α-tubulin (Sigma-Aldrich mAb) staining identifies cilia. Arrows denote a small residual pool of EGFR detectable at the ciliary base of PKD cells. Zeiss LSM510 confocal microscope images (63× objective). Representative results from at least 5 independent experiments. Comparative images are from a single experiment and taken under identical settings. Arrows denote cilia. Scale bar 10 µm. Quantification shows individual ciliary protein intensities normalized to the respective ciliary volume (I–L). Each protein was quantified in 30–100 cilia for each cell type. z-stack images were imported into SlideBook and a volume mask for each cilium was created based on acetylated α-tubulin staining. Staining intensities for each protein were quantified within the respective ciliary volume mask. Statistical evaluation based on two-tailed t-test. (*) p = 0.0105, (***) p<0.0001.

**Figure 7:**
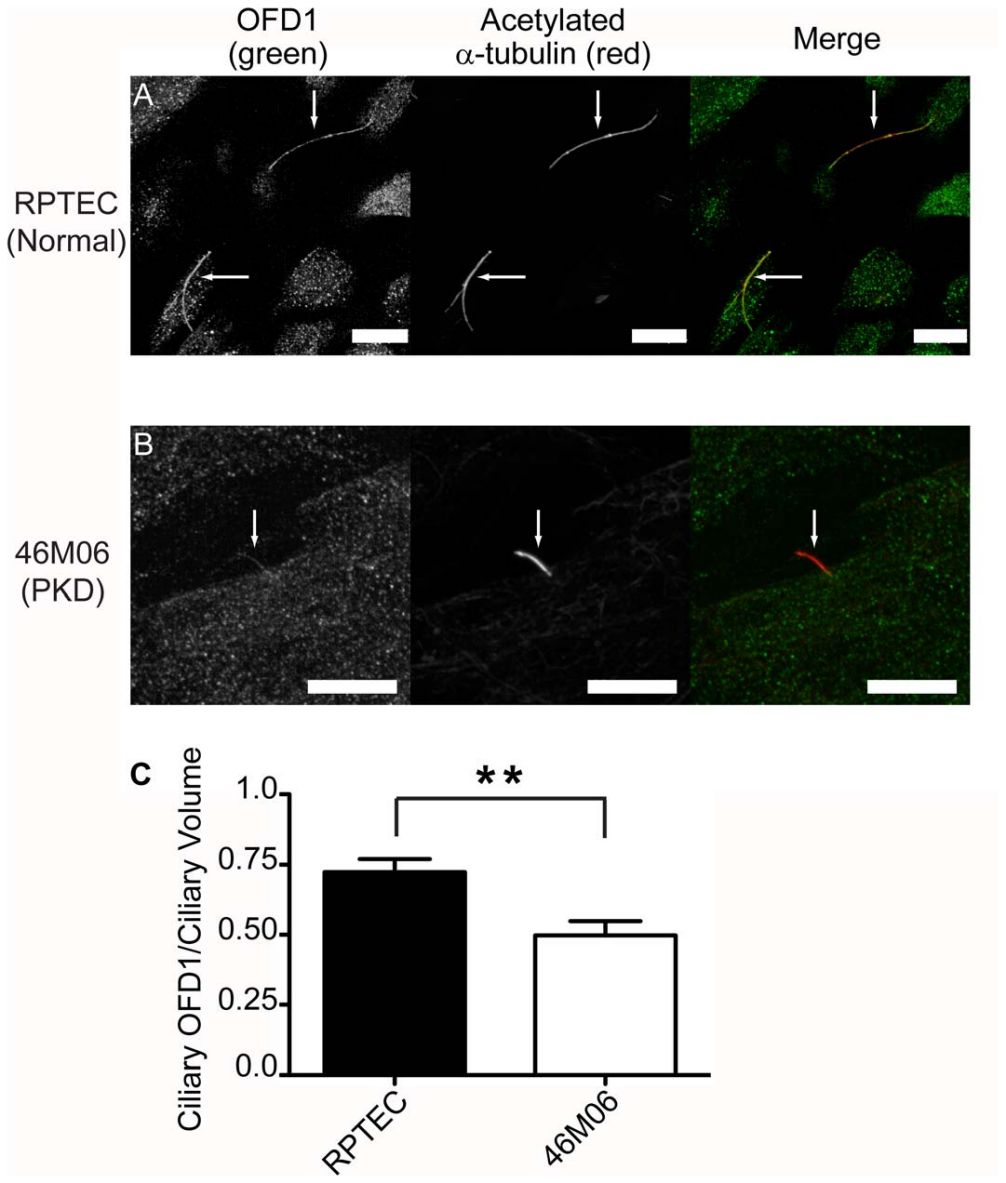
OFD1 ciliary localization is altered in human primary cell lines. Human primary renal proximal tubule epithelial cells (RPTEC) and PKD (44M06) cells were grown on glass coverslips 5 days post confluency to allow for ciliogenesis, fixed and stained using an antibody directed against OFD1 (Santa Cruz pAb). OFD1 localizes to primary cilia in RPTEC cells (A) but is diminished in primary cilia PKD 46M06 cells (B). Acetylated α-tubulin (Sigma-Aldrich mAb) staining identifies cilia. Zeiss LSM510 confocal microscope images (63× objective). Representative results from 3 independent experiments. Comparative images are from a single experiment and taken under identical settings. Arrows denote cilia. Scale bar 10 µm. Quantification shows OFD1 staining intensities normalized to ciliary volumes, performed as detailed in methods and Figure 6 (C). z-stack images were imported into SlideBook and a mask for the cilia was created based on acetylated α-tubulin staining. Statistical evaluation based on two-tailed t-test. (**) p = 0.0036.

Given the shared disease pathologies caused by mutant OFD1 and PKD genes, we tested if the expression of a mutant component of our defined ciliary signaling complex would in turn affect the formation and/or stability of these specialized complexes. In cells from patients with ADPKD, expression of mutant PC1 often results in its absence from primary cilia [31]. Immunolocalization studies were therefore performed on cells from human ADPKD patients with expression of mutant PC1 (Q4004X) [32]. In these cells, there was reduced expression not only of PC1, but also of the associated PC2, EGFR, and flotillin-1 proteins along the shaft of primary cilia (Figure 6A–H). Quantification of multiple data sets verified statistically significant reductions (p<0.0001 for PC1, PC2, and flotillin-2; p = 0.0105 for EGFR) in all components of the complex (Figure 6I–L). A small residual pool of EGFR was sometimes detectable at the ciliary base. OFD1 localization to primary cilia was also significantly decreased in PKD cells as compared to RCTE cells (p<0.0001; data not shown). As further confirmation, we also analyzed OFD1 localization in primary human normal kidney and PKD (46M06) cells. Whilst OFD1 localized to primary cilia of primary normal proximal tubular epithelial cells (RPTEC) cells, it was significantly decreased (P<0.0036) in primary cilia of 46M06 cells (Figure 7B and Figure S3).

**Figure 8:**
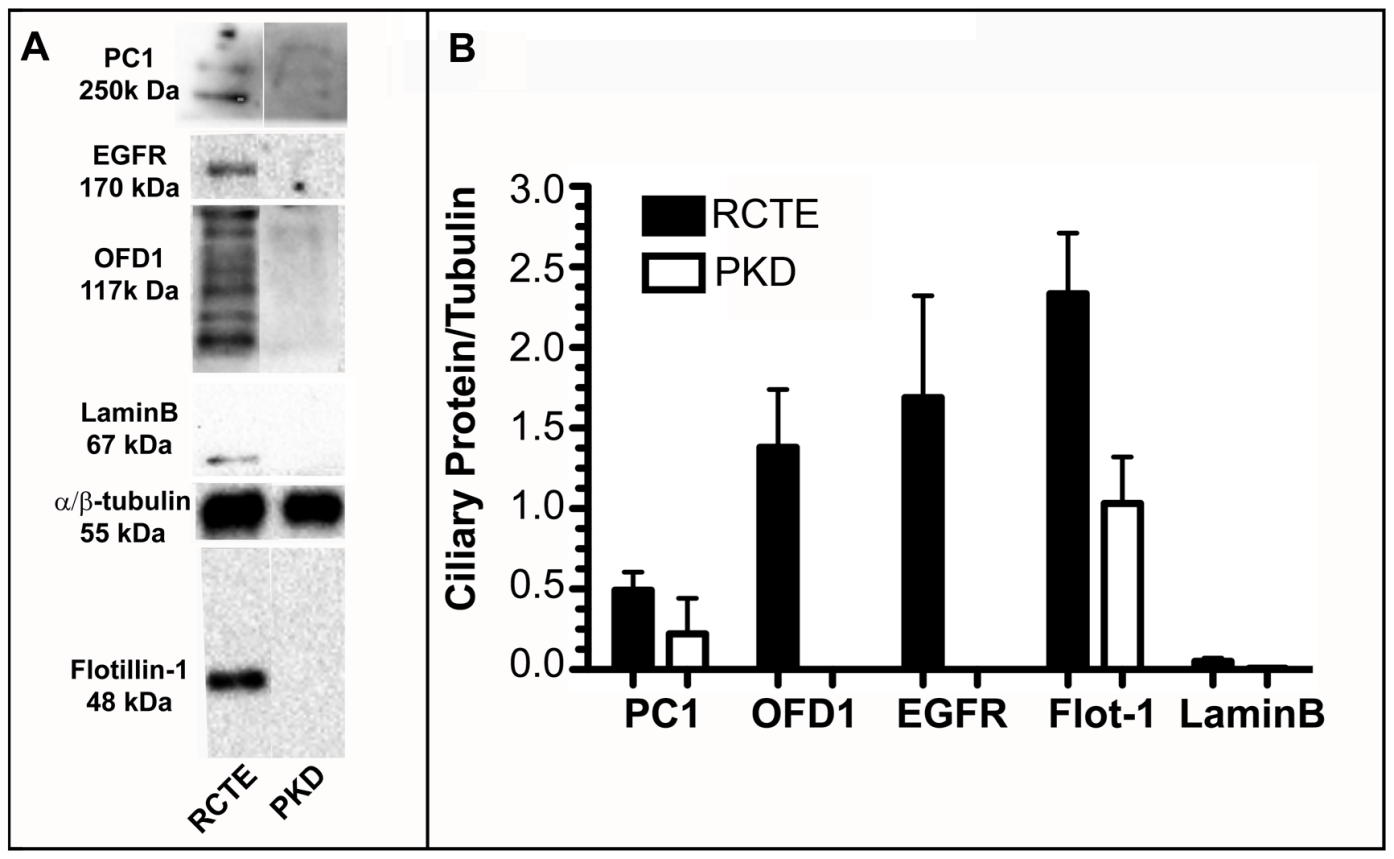
Expression of PC1, OFD1, EGFR, and Flotillin-1 is decreased in ciliary fractions of PKD cells compared to RCTE cells. RCTE cells and PKD Q4004X cells were grown 6 days post-confluency to allow for cell polarization and ciliogenesis and treated with 1.5 mM Dibucaine-HCl for 5 minutes to reduce cell loss and induce shedding of primary cilia. Cilia were collected by fractionation and the ciliary fraction was probed. PC1 (MN032 pAb), OFD1 (Novus Biologicals pAb), EGFR (GeneTex pAb), and flotillin-1 (BD Transduction mAb) were expressed in the ciliary fraction of deciliated RCTE cells but not PKD cells. α/β-tubulin (Cell Signaling pAb) was used as a marker of primary cilia. The nuclear marker lamin B (Santa Cruz pAb) was not present in the ciliary fraction (A). Quantification of 3 independent samples (B). Mean values were found to be statistically different (p<0.0001) using 1-way ANOVA.

Biochemical deciliation experiments were used to independently verify that all components of the identified ciliary complex were decreased in an interdependent manner when polycystin-1 is mutant. Polarized RCTE and PKD (Q4004X) cells were deciliated using Dibucaine-HCL and isolated ciliary fractions analyzed by immunoblot. Quantification of OFD1, EGFR, and flotillin-1 protein levels relative to α/β-tubulin levels confirmed that all components were reduced in the ciliary fraction of the PKD cells as compared to RCTE cells (Figure 8). These data further substantiate the conclusion that the OFD1-EGFR-flotillin complex is lost from cilia in cells with mutant PC1. While siRNA-mediated ablation of OFD1 and PC1 were attempted in renal epithelia and odontoblasts, these studies were complicated by the fact that OFD1 is necessary for ciliogenesis and therefore siRNA knockdown must be performed after cells are fully polarized and have cilia. Under these conditions, a population of OFD1 remained resident in primary cilia even after 48 h of siRNA-mediated knockdown precluding accurate interpretation. The composite data demonstrate that the proteins are organized into a functional complex that is disrupted when polycystin-1 is mutant or absent.

## Discussion

The data presented here identify the presence of a ciliary signaling complex that is conserved between cells of kidney tubules and the oral cavity. We show that this complex consists of the polycystins (PC1 and PC2), the OFD1 ciliopathy disease gene product, the EGFR receptor tyrosine kinase and the membrane raft organizing flotillin proteins. Moreover, we demonstrated that when one of these proteins is mutant it changes the assembly or stability of the entire ciliary signaling complex in the affected cell type, suggestive of co-assembly into a common domain. Indeed, the decreased expression of OFD1 in primary cilia when PC1 is mutant offers a molecular basis for some of the common pathologies of OFD and ADPKD.

There are two molecular explanations for the finding that mutant PC1 results in reductions of OFD1 and other key signaling proteins in primary cilia that immediately come to mind. First, PC1 and OFD1 may have interdependent roles in the ciliary transport and/or targeting of the proteins in the complex. Second, PC1 may be essential for stabilizing the complex within the ciliary membrane. Based on the fact that the levels of OFD1, PC2 and flotillins were more significantly affected than EGFR, it is possible that OFD1, PC2 and the flotillins are highly dependent on functional PC1 for ciliary delivery and stabilization whereas EGFR may arrive at the primary cilium independently of PC1 but relies on functional PC1 for integration into ciliary signaling protein assemblies. During cell division OFD1 localizes to centrosomes, and later to basal bodies and the shaft of primary cilia in polarized differentiated cells. Thus, OFD1 like the polycystins may have multiple functions that are tied to different locations and protein-protein interactions [2], [3], [33].

OFD1 functions at the base of cilia to control ciliogenesis through interactions with intraflagellar transport and possibly vesicle docking machineries. The functional importance of OFD1 in recruitment of IFT components is reflected in that the loss of either OFD1 or IFT88 results in similar phenotypes [4], [8]–[10]. The coiled-coil domains 2 through 4 of OFD1 interact with IFT88 and recruit IFT particles to the distal appendages of centriolar satellites, which are necessary for initiation of ciliogenesis [12]. New studies indicate autophagy is subsequently required for the selective removal of OFD1 from centriolar satellites to allow ciliogenesis to proceed [34]. Distal appendages are also implicated in the trafficking and docking of ciliary vesicles carrying membrane constituents [35]–[38]. If ciliary vesicle docking at the distal appendages requires an interaction between PC1 and OFD1, it is conceivable that the ciliary localizations of multiple components of a preassembled complex would be similarly affected. The C2 calcium-dependent domain containing 3 (C2cd3) protein is another essential regulator of primary cilium biogenesis that localizes to distal appendages, though how C2cd3 is integrated with OFD1 regulated ciliogenesis remains to be dissected [39]. Thus, the diminished OFD1 localization to cilia when PC1 is mutant may adversely affect both ciliogenesis and ciliary function based on the dual roles OFD1 has in regulating intraflagellar transport components and membrane vesicles.

The fact that loss of ciliary PC1 led to decreased OFD1 localization to the ciliary shaft suggests that polycystins might also serve to promote stable association of OFD1 with the ciliary membrane. OFD1 contains five coiled coil domains [5]; and it is possible that these domains have roles beyond enabling the distal appendage localization of OFD1. For example, interactions with coiled coil domains in the polycystins might mediate delivery to and stable localization in the ciliary shaft [3]. Support for this speculation is provided by previous reports showing PC1 and PC2 also contain CC domains that mediate their interaction, and are important for their ciliary localization [40], [41]. Further characterizing the molecular basis for the interaction between membrane proteins (such as polycystins) and soluble proteins (such as OFD1) at the distal appendages and within the ciliary shaft will be important to distinguish the role(s) of polycystin-1 in complex transport and/or stabilization.

Our demonstration that the flotillins localize to primary cilia of renal epithelia and odontoblasts and flotillin-2 interacts with the polycystins, EGFR and OFD1 in both cell types is extremely exciting for several reasons. The presence of the flotillins in cilia supports previous speculation regarding commonalities between basolateral membrane, immune synapse and cilia membrane organization [6], [42], [43]. Flotillins are important in the formation of ordered membrane domains in hematopoietic cells where they are critical for cell polarization and lymphoid immune synapse formation [18], [44], [45]. In polarized Madin-Darby canine kidney cells, both flotillin-1 and -2 have a non-polarized distribution. Additionally, the flotillins were suggested to have a role in polarization that is restricted to hematopoietic cells [44]. However, as we showed in human renal epithelial cells flotillins bind cholesterol and organize the polycystins, E-cadherin, tyrosine kinases and phosphatases at the lateral membrane [20]. As shown in this work, flotillins may also organize the polycystins, EGFR and OFD1 in primary cilia into specialized domains. The fact that ciliary EGFR levels were was less compromised than the other components is interesting and suggests that the tyrosine kinase may move in and out of cholesterol based raft domains organized by flotillins and that this is likely to have functional importance in regulating ciliary signaling.

Flotillins play a major role in the fidelity of cell signaling and EGFR function [46]–[48]. Knockdown of flotillin-2 disrupts localization and phosphorylation of EGFR and activation of downstream MAPK signaling components [22]. However, in breast cancer cells with mutant PIK3CA, a well-known downstream target of EGFR signaling, knockdown of flotillin-1 causes an upregulation of EGFR and hyperactivation of MAPK signaling. These data indicate that the flotillins have dual roles in enabling receptor tyrosine kinase activation and downstream signaling, as well as in restraining signaling components such as PIK3CA in an inactive state [49]. The importance of ciliary organization of EGFR signaling is further reinforced by recent studies showing that when cilia are ablated, EGFR mediated activation of apical calcium channels is increased 64-fold [50]. Similarly, in inner medullary collecting duct cells (IMCD-3) primary cilia were found to restrain interaction and cross-talk between G-protein coupled receptors, which are also known to cross-talk with receptor tyrosine kinases [51], [52]. Hence, it appears likely that the flotillins have an important role in the control of ciliary signaling that extends beyond a purely scaffolding function. Notably, the upshift in molecular weight of flotillin proteins in the complex is suggestive of post-translational modifications that may be important in their regulation of specific signaling events.

In addition to EGFR, there are growing numbers of receptor tyrosine kinases identified in primary cilia of epithelia, neuronal cells and fibroblasts [52]. Of particular interest are the expression of RON kinase in motile cilia of airway epithelia and the documented cooperation between EGFR, RON and Met [53]–[55]. Emerging data from studies in human embryonic kidney cells and in breast, lung and colorectal cancer cells indicate that the RON kinase interacts with EGFR and may contribute to direct transcriptional regulation [56], [57]. The connections between RON, Met and EGFR are intriguing because both Met and EGFR are pivotal in cystogenesis [58]–[60]. Counterbalancing the activities of receptor tyrosine kinases are receptor protein phosphatases, with members of the LAR family shown to be present in cilia of renal epithelia [21]. Additional regulation may occur at the level of proteasomal degradation at the ciliary base [57]. Therefore, the present studies lay the groundwork for how receptor tyrosine kinases may be organized in cilia and warrant an expanded characterization to determine how receptor protein kinases and phosphatases along with other signaling proteins may interact with or be co-assembled with the flotillin-EGFR-polycystin-OFD1 protein complexes characterized here.

In sum, our findings of a protein complex containing the polycystins, flotillins, EGFR and OFD1 in cilia of both renal and dental cells provides evidence for the existence of ciliary signaling microdomains. The requirement for the integrity of the complex provides a plausible molecular rationale for the commonalities between craniofacial disorders and cystic kidney disease and informs strategies for the development of potential therapeutic interventions that stabilize protein complexes.

## Materials and Methods

### Cells and Reagents

Dental pulp-derived odontoblasts were used as representative neural crest derived cells from the oral cavity. The previously characterized MO6-G3 immortalized mouse dental pulp-derived odontoblast cell line was used as described [61]. MO6-G3 cells were grown in alpha-MEM supplemented with 10% FCS, 100 units/ml penicillin and streptomycin, 50 µg/ml ascorbic acid at 33°C in a humidified atmosphere of 95% air and 5% CO_2_, with media changes every two days. The previously characterized PKD Q4004X PKD cells [32] were a generous gift from Dr. Robert Bacallao (Indiana University School of Medicine, Indianapolis, IN). Primary human cell line 46M06 was obtained from renal cysts of an ADPKD kidney. Human cell lines from renal cortical tubular epithelia (RCTE) were immortalized and cultured as previously described (Nauli, 2006, Ward 2011). Renal proximal tubule cells (RPTEC) were purchased from Lonza (Walkersville, MD) and cultured using RCTE conditions. Cell culture reagents were purchased from Invitrogen/Gibco (Carlsbad, CA). Primary antibodies were purchased from the following vendors: mouse mAb directed against acetylated α-tubulin (Sigma-Aldrich T7451, St. Louis, MO); rabbit pAb directed against α/ß-tubulin (Cell Signaling Technology #2148, Danvers, MA); mouse mAb anti-actin, clone C4 (Millipore #MAB1501, Temecula, CA); goat pAb directed against Lamin B (Santa Cruz sc-6217, Santa Cruz, CA); rabbit pAb directed against PC1 NM005 raised by immunizing rabbits with a distal carboxy-terminal fragment of polycystin-1 (amino acids 4070-4302) (Roitbak, 2004); rabbit anti-PC1 NM002 and NM032 raised in rabbits immunized with a peptide corresponding to aa 3633–3645 of human PC1, and with an N-terminal cysteine (CKRLHPDEDDTLVE; Suzanna Horvath, California Institute of Technology, Pasadena, CA). The peptide was conjugated to keyhole limpet hemocyanin using benzoquinone and was used to immunize rabbits NM002 and NM032 (Covance, Denver, PA). For affinity purification, the same peptide was conjugated to Sulfalink gel (#20401; Pierce) according to the manufacturer's instructions (1 mg peptide/1 ml resin bed volume). Ten milliliters NM002 serum was incubated with 5 ml resin and washed to remove unbound immunoglobulin and serum proteins. Bound immunoglobulin was eluted with 0.1 M glycine (pH 2.5), immediately neutralized with Tris base, and used for immunoblotting and immunostaining experiments at a concentration of 10 µg/ml. (Ward, 2011); mouse mAb anti-polycystin-1 (Santa Cruz Biotechnology sc-130554, Santa Cruz, CA); rabbit pAb anti-polycystin-2 (AbCam ab78622, Cambridge, MA); rabbit pAb anti-polycystin-2 (Santa Cruz Biotechnology sc25749, Santa Cruz, CA); rabbit pAb anti-EGFR (GeneTex GTX121919, Irvine, CA); rabbit pAb anti-EGFR (Santa Cruz Biotechnology sc-03, Santa Cruz, CA); rabbit pAb anti-ErbB2 (US Biological E3451-27, Swampscott, MA); rabbit mAb anti-ErbB2 (Epitomics #2064-1, Burlingame, CA); rabbit pAb anti-OFD1 (Novus Biologicals NBP1-89355, Littleton, CO); goat pAb anti-OFD1 (Santa Cruz Biotechnology sc-168837, Santa Cruz, CA); rabbit pAb anti-Flotillin-1 (AbCam ab-50671, Cambridge, MA); mouse mAb anti-Flotillin-1 (BD Transduction Laboratories 610820, San Jose, CA); rabbit mAb anti-Flotillin-2 (Cell Signaling Technology #3436, Boston, MA); mouse mAb anti-Flotillin-2 (BD Transduction Laboratories 610383, San Jose, CA); ECL Anti-Rabbit IgG-HRP (GE Healthcare NA934V, Little Chalfont, Buckinghamshire); ECL Anti-Mouse IgG-HRP (GE Healthcare NA931V, Little Chalfont, Buckinghamshire); donkey anti-goat IgG-HRP (Santa Cruz sc-2020, Santa Cruz, CA); Rabbit TrueBlot ULTRA: Anti-Rabbit Ig HRP (eBioscience 18–8816, San Diego, CA); Mouse TrueBlot ULTRA: Anti-Mouse Ig HRP (eBioscience 18–8817, San Diego, CA); mouse IgG2a, κ mouse isotype control (Sigma-Aldrich M5409, Saint Louis, MO) normal rabbit IgG isotype control (Santa Cruz Biotechnology sc2027, Santa Cruz, CA). The following fluorophore-conjugated secondary antibodies (Alexa Fluor dyes) were used for immunofluorescence assays: Alexa Fluor 488 Donkey Anti-Mouse IgG (Invitrogen A-21202, Grand Island, NY); Alexa Fluor 488 Donkey Anti-Rabbit IgG (Invitrogen A-21206, Grand Island, NY); Alexa Fluor 555 Donkey Anti-Mouse IgG (Invitrogen A-31570, Grand Island, NY); Alexa Fluor 488 Donkey Anti-Goat IgG (Invitrogen A-11055, Grand Island, NY); Alexa Fluor 555 Donkey Anti-Rabbit IgG (Invitrogen A-31572, Grand Island, NY); Alexa Fluor 647 Donkey Anti-Mouse IgG (Invitrogen A-31571, Grand Island, NY); Alexa Fluor 647 Donkey Anti-Rabbit IgG (Invitrogen A-31573, Grand Island, NY); Alexa Fluor 647 Donkey Anti-Goat IgG (Invitrogen A-21082, Grand Island, NY).

### Immunofluorescence Staining

For ciliary immunolocalization experiments, cells were grown on glass coverslips or 0.4 µm filter supports (Falcon-BD 353090, Franklin Lakes, NJ) for 4–6 days post-confluence and fixed with 3% paraformaldehyde (PFA) and processed for immunostaining as previously described [6]. Briefly, cells were permeabilized using 0.1% Triton-X 100 in 0.2% cold fish gelatin (blocking agent), and primary and secondary antibody incubations were performed in a humidified chamber at 37°C. Cells were labeled with antibodies directed against OFD1, EGFR, ErbB2, PC1, PC2, or flotillin-1. Cells were co-labeled with anti-acetylated α-tubulin or anti-α/β-tubulin to identified primary cilia. Controls for specificity and auto-fluorescence included staining with secondary antibodies alone. Ciliary colocalization of proteins with acetylated α-tubulin was defined as exhibiting colocalization of the two signals across at least two pixels within the cilium as imaged in the confocal Z-stack data. Confocal immunofluorescence images were collected using a Zeiss LSM510 or Zeiss LSM 510-META laser-scanning confocal microscope (Carl Zeiss, Thornwood, NY) with 40x, 1.3 numerical aperture (NA) or 63x, 1.4 NA oil immersion objectives. LSM 510 Image Acquisition software (Carl Zeiss) was used to acquire images. Confocal Z-stacks were processed with the Zeiss Image Browser or Voxx2 (provided freely for noncommercial use by the Indiana Center for Biological Microscopy, Indianapolis, IN, www.nephrology.iupui.edu/imaging/voxx/index.htm), and assembled using Adobe Photoshop and Illustrator (Adobe, San Jose, CA).

Quantification was performed by importing z-stack images (all taken at identical settings) into SlideBook software (Intelligent Imaging) and creating a ciliary mask based on acetylated α-tubulin staining. Ciliary marker (OFD1, PC1, PC2, flotillin-1 and EGFR) staining intensities within the ciliary mask were quantified and normalized based on ciliary volume. At least 30–100 cilia were quantified from multiple experiments.

Movies were generated using the movie maker feature in Voxx2 software, and converted to .avi and .mpg file formats using QuickTime Player (Apple, Cupertino, CA) and TMPGEnc (Pegasys, Tokyo, Japan), respectively.

### Transient transfections with flotillin-2-GFP or GFP-OFD1

Flotillin-2–EGFP (where EGFP stands for enhanced green fluorescent protein) encoding full-length rat reggie-1 fused in frame at the C-terminus to EGFP was provided by Dr. Ritva Tikkanen [47]. GFP-OFD1 encoding full-length OFD1 fused in frame at the N-terminus to pEGFP-N1 vector [3] was provided by Dr. Andrew Fry. Cells were plated at confluency on filter supports and cultured as described above 4–6 d post-confluency to ensure ciliogenesis. Transfections were performed by electroporation (iPoration-Primax Biosceinces, Menio Park, CA) according to the manufacturer's instructions. The iPorator (Primax Biosciences, Inc.) yields 50–60% transfection efficiency in both cell types (Figure S4). Flotillin-2-GFP and GFP-OFD1 transfection efficiency of ∼40% was consistently observed. Cells were fixed with 3% PFA 16 h post-transfection, and viewed directly or processed for co-immunostaining.

### Proximity Ligation Assay (PLA)

PCR-based visualization was performed using Duolink II Rabbit/Mouse Red Kit (Olink Bioscience #92101) following manufacturer's instructions. Primary antibodies used were: rabbit anti-EGFR (GeneTex, Irvine, CA) and anti-polycystin-1 (Santa Cruz Biotechnology, Santa Cruz, CA).

### Immunoprecipitations

Cells were grown 3–5 days post-confluency to ensure cilial formation. Cells were washed in PBS and lysed on ice in lysis buffer (0.5% NP40, 10 mM Tris-HCL pH 7.4, 150 mM NaCl, 5 mM EDTA, 50 mM NaF, 1 mM Na_3_VO_4_, 60 mM n-Octyl-β-D-glucopyranosid (AppliChem A1010, Saint Luios, MO), supplemented with protease inhibitor cocktail (Calbiochem/EMD Chemicals, Gibbstown, NJ). Lysates were precleared by centrifugation at 10,000 rpm in an Eppendorf microcentrifuge for 10 minutes and pretreated with protein G Sepharose or protein A Sepharose (Amersham Biosciences/GE Healthcare, Piscataway, NJ); 500 µg of total protein was used for immunoprecipitations. 500 µg cell lysate/40 µg protein A Sepharose or protein G Sepharose at 4°C for 3 hours on a rotator. Precleared lystate supernatant was transferred to a clean microfuge tube and incubated with 1–3 µg of antibody directed against specified protein at 4°C for 2 hours with gentle rotation. Antibody complexes were recovered by incubation with Protein G or Protein A Sepharose beads at 4°C overnight. The immunoprecipitates were washed three times in lysis buffer and supplemented with SDS-PAGE loading buffer, heated for 5 minutes at 94°C, and resolved by SDS gel electrophoresis.

### Deciliation Assay

Renal epithelial cells were deciliated using 1.5 mM dibucaine-HCL for 5 minutes or 5 mM dibucaine-HCL in culture media for 5 minutes or 15 minutes, both of which rapidly and selectively caused the intact shedding of primary cilia from the cell surface as was observed previously for IMCD3 cells [62], [63]. Dibucaine treatment conditions were optimized by testing different concentrations and treatment times with the goal of promoting cilial release without cell detachment. A stock solution of dibucaine HCl (25 mM) was diluted to 1.5 mM or 5 mM in Tyrodes balanced salt solution. RCTE and MO6-G3 cells were grown on 60 mM collagen-1 coated cell culture dishes for 5–6 days post-confluency to allow for ciliogenesis. Media was aspirated and cells were washed three times in PBS+. Dibucaine was then added for 5–15 minutes with agitation causing deciliation. Tyrodes/dibucaine-HCl solution containing the cilia were then collected and centrifuged at 850 g_max_ for 10 minutes to collect cell bodies. The supernatant containing the ciliary fraction was collected and cilia were harvested by centrifugation at 28,000 g_max_ for 30 minutes. Supernatant was removed and pellet containing ciliary fraction was resuspended using 30 µl of lysis buffer and run on a SDS-PAGE gel.

### Statistical Analysis

For biochemical assays, means and standard errors were used to summarize the data from each experiment. For immunofluorescence experiments a minimum of 30–100 cells/condition were counted. All experiments were repeated at least three times. For immunoprecipitations comparisons across groups were made using One-way analysis of variance (ANOVA) to determine whether the group means were significantly different. When significant differences in group means are found, post-hoc multiple comparison tests (e.g., Tukey's test) were used to determine which groups are significantly different from one another. For protein expression analyses and immunofluorescence quantifications differences were analyzed by unpaired, two-tailed Student's *t* test. GraphPad Prism version 5.0 was used to perform statistical analyses.

## Supporting Information

Figure S1 **Two Protein Co-Localization in Primary Cilia of RCTE.** Flotillin-2 was transiently transfected into polarized RCTE and cells and then fixed and stained using the AbCam pAb against PC2 (A) or the GeneTex pAb against EGFR (B), Polarized RCTE cells were fixed and stained using the GeneTex pAb against EGFR and NM002 pAb against PC1 (C). Acetylated α-tubulin (Sigma-Aldrich mAb) labeling identifies cilia. Zeiss LSM510 confocal microscope images (63× objective). Arrows denote the protein of interest within a cilium. Representative results from at least 3 independent experiments. Secondary antibody only controls were negative (not shown). Scale bar 10 µm.
(TIF)

Figure S2 **Deciliation of RCTE cells.** Polarized renal epithelial cells were treated with 5 mM Dibucaine-HCl for 15 minutes causing the cells to shed their primary cilia. Cilia were collected by fractionation and the ciliary fraction was probed. PC1 (NM032 pAb), PC2 (Santa Cruz pAb), and EGFR (Santa Cruz pAb) were expressed in the ciliary fraction of deciliated RCTE cells. The nuclear marker lamin B (Santa Cruz pAb) was not present in the ciliary fraction. Representative results from 4 independent experiments. α/β-tubulin was used as a marker of primary cilia.
(TIF)

Figure S3 **OFD1 Reduced in Primary Cilia of PKD 46M06 Cells.** Polarized 46M06 PKD cells were fixed and stained using an antibody directed against OFD1 (Novus Biologicals pAb). Acetylated α-tubulin (Sigma-Aldrich mAb) labeling identifies cilia. 63× objective with no zoom (A), 63× zoomed in image of panel A cilia marked with arrows (B), 63× zoomed in images of separate fields of view (C–D). Zeiss LSM510 confocal microscope images. Representative results from at least 3 independent experiments. Secondary antibody only controls were negative (not shown). Scale bar 10 µm.
(TIF)

Figure S4 **iPoration yields high transfection efficiencies for plasmid expression vectors and siRNA in post-confluent, polarized MO6-G3 and RCTE cells.** MO6-G3 odontoblasts are an inherently more difficult cell line to transfect. This problem is compounded further by the necessity to transfect these cells after they are 100% confluent and have a completely polarized phenotype with very tight cell-cell contacts. With iPoration transfection efficiencies were 60–70% for FITC-labeled siRNA and 40–60% for flotillin-2-GFP in both MO6-G3 and RCTE cell types. As a comparison, traditional lipofection using Lipofectamine 2000 (Life Technologies) resulted in transfection efficiencies that were consistently <10%.
(TIF)

Movie S1 **PC1 and EGFR interact within primary cilia of RCTE cells.** Polarized RCTE cells were grown on coverslips 5 days post-confluency. Cells were incubated with antibodies against PC1 (Santa Cruz mAb) and EGFR (GeneTex pAb), followed by Duolink PLA Probes to identify points of PC1-EGFR interaction (red). Cilia identified by acetylated α-tubulin (green). Movie generated using Voxx2 (provided freely for noncommercial use by the Indiana Center for Biological Microscopy, Indianapolis, IN, www.nephrology.iupui.edu/imaging/voxx/index.htm). Scale bar is 5 µm.
(MPG)
