# Supplementary figures and images for: OFD1 and Flotillins Are Integral Components of a Ciliary Signaling Protein Complex Organized by Polycystins in Renal Epithelia and Odontoblasts
Source: PLoS One. 2014 Sep 2;9(9):e106330. doi: 10.1371/journal.pone.0106330 (PMC4152239; doi:10.1371/journal.pone.0106330)

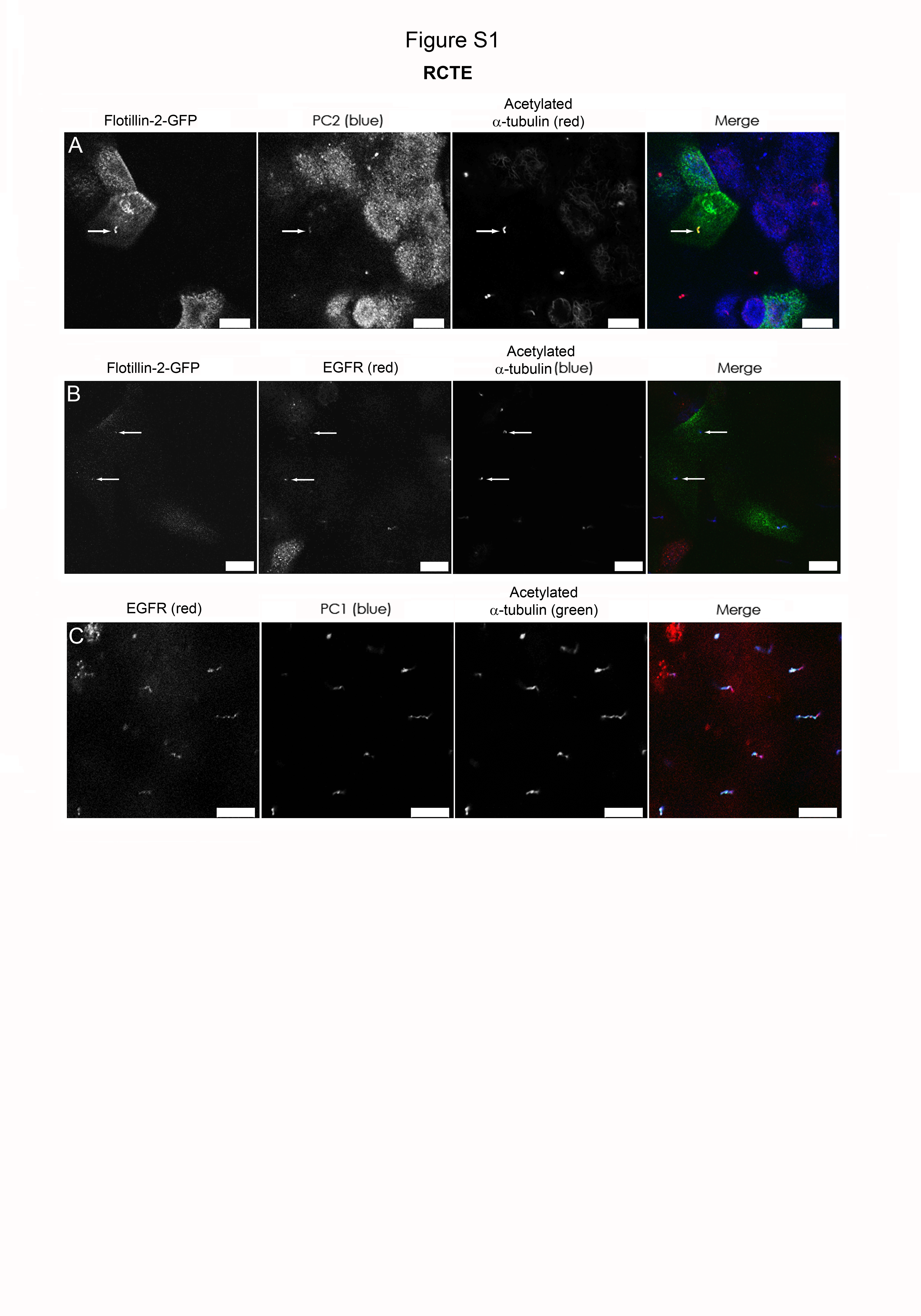

Supplement: Figure S1 — Two Protein Co-Localization in Primary Cilia of RCTE. Flotillin-2 was transiently transfected into polarized RCTE and cells and then fixed and stained using the AbCam pAb against PC2 (A) or the GeneTex pAb against EGFR (B), Polarized RCTE cells were fixed and stained using the GeneTex pAb against EGFR and NM002 pAb against PC1 (C). Acetylated α-tubulin (Sigma-Aldrich mAb) labeling identifies cilia. Zeiss LSM510 confocal microscope images (63× objective). Arrows denote the protein of interest within a cilium. Representative results from at least 3 independent experiments. Secondary antibody only controls were negative (not shown). Scale bar 10 µm. (TIF) [file pone.0106330.s001.tif]

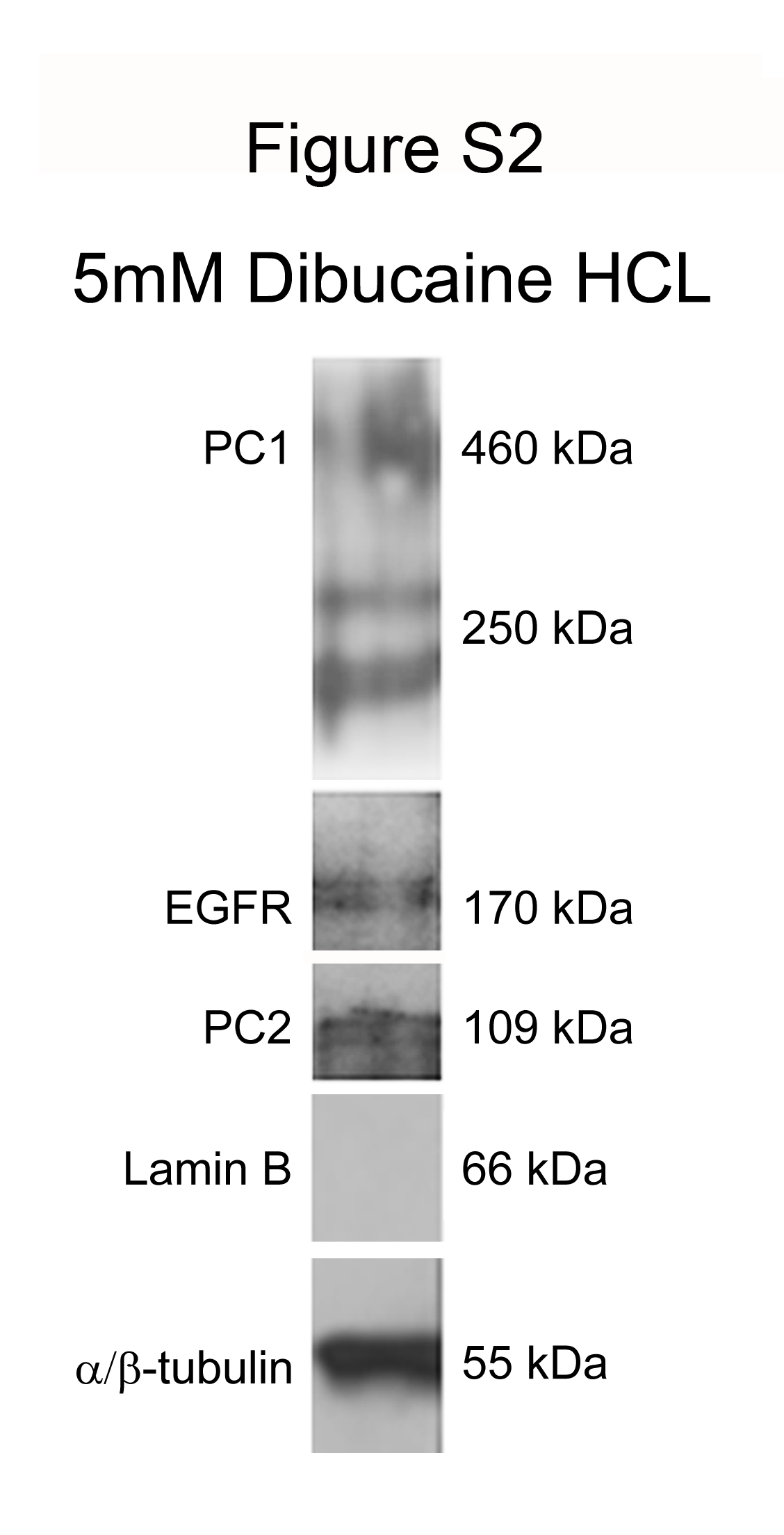

Supplement: Figure S2 — Deciliation of RCTE cells. Polarized renal epithelial cells were treated with 5 mM Dibucaine-HCl for 15 minutes causing the cells to shed their primary cilia. Cilia were collected by fractionation and the ciliary fraction was probed. PC1 (NM032 pAb), PC2 (Santa Cruz pAb), and EGFR (Santa Cruz pAb) were expressed in the ciliary fraction of deciliated RCTE cells. The nuclear marker lamin B (Santa Cruz pAb) was not present in the ciliary fraction. Representative results from 4 independent experiments. α/β-tubulin was used as a marker of primary cilia. (TIF) [file pone.0106330.s002.tif]

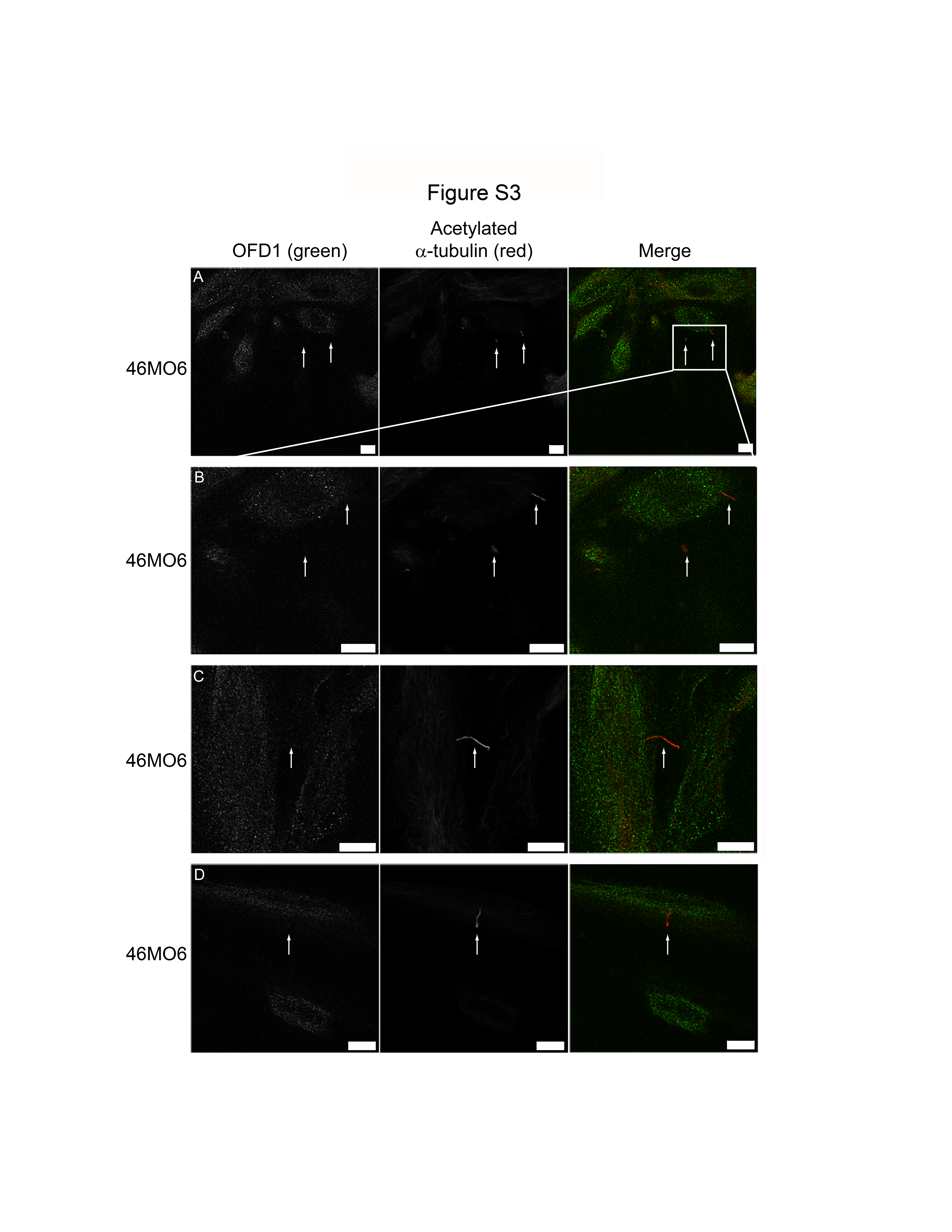

Supplement: Figure S3 — OFD1 Reduced in Primary Cilia of PKD 46M06 Cells. Polarized 46M06 PKD cells were fixed and stained using an antibody directed against OFD1 (Novus Biologicals pAb). Acetylated α-tubulin (Sigma-Aldrich mAb) labeling identifies cilia. 63× objective with no zoom (A), 63× zoomed in image of panel A cilia marked with arrows (B), 63× zoomed in images of separate fields of view (C–D). Zeiss LSM510 confocal microscope images. Representative results from at least 3 independent experiments. Secondary antibody only controls were negative (not shown). Scale bar 10 µm. (TIF) [file pone.0106330.s003.tif]

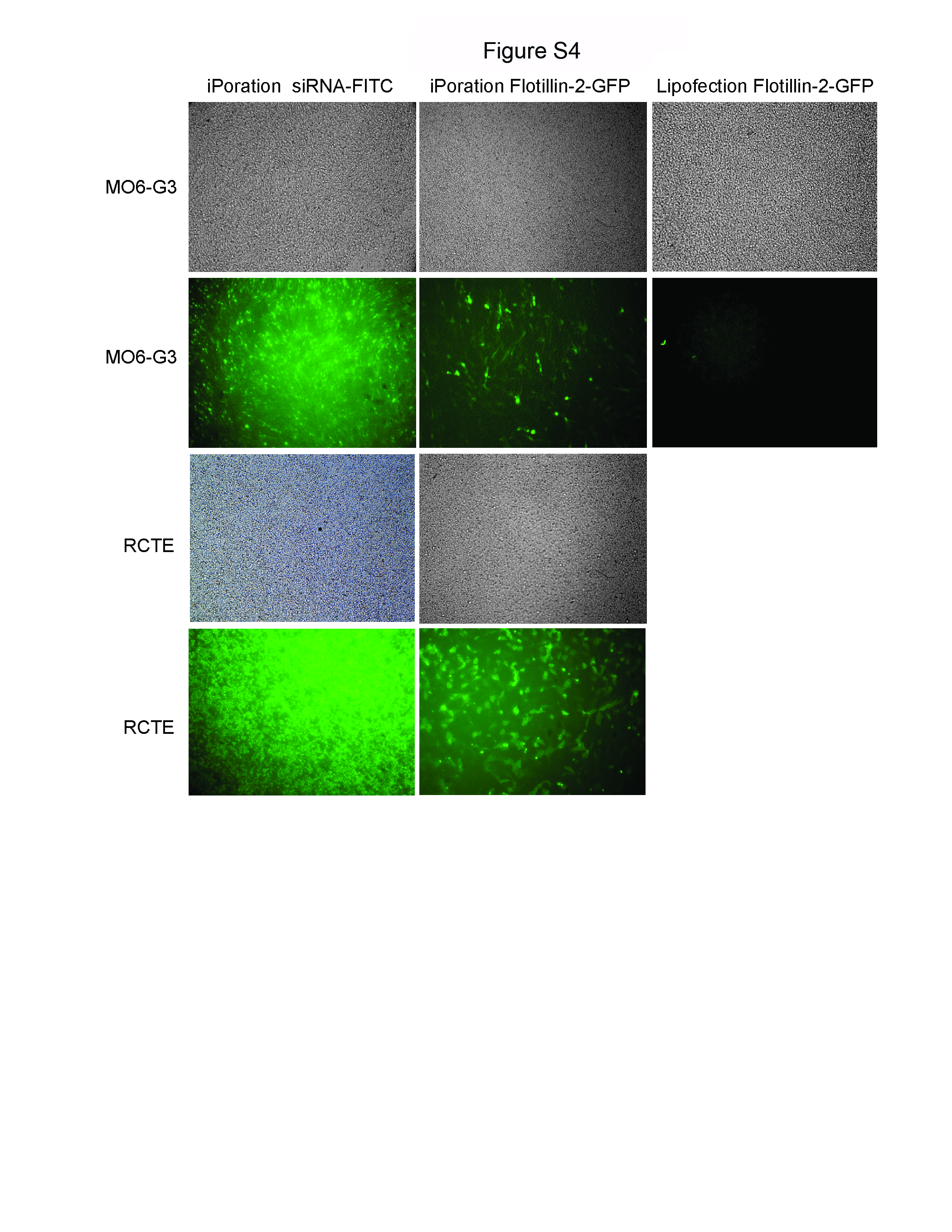

Supplement: Figure S4 — iPoration yields high transfection efficiencies for plasmid expression vectors and siRNA in post-confluent, polarized MO6-G3 and RCTE cells. MO6-G3 odontoblasts are an inherently more difficult cell line to transfect. This problem is compounded further by the necessity to transfect these cells after they are 100% confluent and have a completely polarized phenotype with very tight cell-cell contacts. With iPoration transfection efficiencies were 60–70% for FITC-labeled siRNA and 40–60% for flotillin-2-GFP in both MO6-G3 and RCTE cell types. As a comparison, traditional lipofection using Lipofectamine 2000 (Life Technologies) resulted in transfection efficiencies that were consistently <10%. (TIF) [file pone.0106330.s004.tif]
